# Tumor-associated lymphangiogenesis predicts unfavorable prognosis of intrahepatic cholangiocarcinoma

**DOI:** 10.1186/s12885-019-5420-z

**Published:** 2019-03-08

**Authors:** Meng Sha, Seogsong Jeong, Xin Wang, Ying Tong, Jie Cao, Han-Yong Sun, Lei Xia, Ning Xu, Zhi-Feng Xi, Jian-Jun Zhang, Xiao-Ni Kong, Qiang Xia

**Affiliations:** Department of Liver Surgery, Ren Ji Hospital, School of Medicine, Shanghai Jiao Tong University, 1630 Dongfang Road, Shanghai, 200127 China

**Keywords:** Tumor-associated lymphangiogenesis, Intrahepatic cholangiocarcinoma, Prognosis, Overall survival, Recurrence-free survival

## Abstract

**Background:**

Tumor-associated lymphangiogenesis is considered significant in number of solid malignancies. However, its impact on prognosis of intrahepatic cholangiocarcinoma (ICC) after resection remains further confirmation. Herein, we conducted this study to evaluate prognostic impact of tumor-associated lymphangiogenesis in patients with ICC.

**Methods:**

Extent of tumor-associated lymphangiogenesis of ICC was evaluated by quantifying microlymphatic vessel density (MLVD) from immunohistochemical staining of a lymphatic endothelial-specific antibody (podoplanin). Clinicopathological characteristics were comprehensively analyzed to identify MLVD-associated factors. The patients were stratified into high and low MLVD groups according to the distinctive correlation between the MLVD and overall survival using the Spearman’s correlation test. Kaplan-Meier estimation was performed to confirm prognostic impact of MLVD in patients with ICC. Univariate and multivariate analyses were performed using the Cox proportional hazard model.

**Results:**

The MLVD between 4 to 12 counts showed inverse proportion to the overall survival (Spearman’s r = − 0.66; 95% confidence interval [CI], − 0.82 to − 0.39; *p* <  0.0001), which was set as a cut-off for the high MLVD group, whereas the MLVD between 13 to 25 showed no correlation to the overall survival (r = − 0.11; 95% CI, − 0.38 to 0.19; *p* = 0.4791). The high MLVD group showed more frequent lymph node metastasis (*p* <  0.001) and were more likely to suffer from recurrence of the tumor compared to the low MLVD group (*p* <  0.001). The high MLVD was found to be independently associated with reduced overall and recurrence-free survival. The 5-year overall survival of the patients with high MLVD was significantly lower compared to those with low MLVD (0% vs 48%).

**Conclusions:**

Our study reveals that tumor-associated lymphangiogenesis is significantly associated with increased lymphatic metastasis, recurrence of the tumor, and reduced overall survival in patients with ICC, thus providing guidance when estimating postresection prognosis.

## Background

Intrahepatic cholangiocarcinoma (ICC) is the second most common primary liver cancer that accounts for approximately 10 to 15% of all primary liver malignancies [1, 2]. Radical resection of the tumor is the potential curative treatment that prolongs survival outcomes [3]. However, most patients lose the opportunity to undergo radical surgery due to advanced stage or extrahepatic metastasis at the time of diagnosis [4]. Moreover, application of adjuvant therapies, such as chemotherapy and radiotherapy, is yet to be conclusively confirmed whether these provide significantly better survival outcomes [5]. In consideration of increasing number of patients and limited therapeutic strategies, a better understanding of potential risk factors of ICC is essential to seek novel anti-tumor targets and to develop new treatment approaches.

Metastasis of tumor cells is one of the leading causes of mortality in ICC and occurs mainly through lymphatic vessels [6]. In recent years, increasing experimental and clinicopathological studies indicated that lymphatic vessels undergo dynamic changes within the tumor, including tumor-associated lymphangiogenesis, that facilitate metastasis [7]. Specific signaling pathways, such as vascular endothelial growth factor C (VEGF-C) and its cognate receptor VEGF receptor 3 (VEGFR-3) are confirmed to stimulate tumor-associated lymphangiogenesis, as well as lymph node metastasis in experimental tumor models [8–10]. Apart from that, lymphangiogenesis was found to be associated with increased frequency of tumor cells in lymphatics and lymph nodes in hilar cholangiocarcinoma [11]. However, information regarding the role of tumor-associated lymphangiogenesis in progression and prognosis of ICC remains poor. In this study, we evaluated impact of tumor-associated lymphangiogenesis in patients with ICC, a highly malignant and aggressive primary liver cancer.

## Methods

### Patients and cancer tissues

A total of 106 patients from Jan 2007 to Jul 2015 who underwent hepatic resection with curative intent for ICC were enrolled into the present study. The patients met the criteria as we described before [12]. Briefly, patients who were pathological confirmed of ICC without distant metastasis were included. The present study was applied by the local ethics committee of Renji Hospital, Shanghai Jiao Tong University. Consents from individual patients were waived due to retrospective nature of the study.

### Data collection

All patients were followed up in the outpatient at regular intervals and those with unavailable data were visited by telephone inquiries or dropping-in follow up. The primary endpoint of this study was death of patients, and the second endpoint was recurrence of the tumor. Data of last follow-up investigation and recurrence of the tumor were collected for all patients.

### Immunohistochemistry and assessment of micro-lymphatic vessel density

To analyze tumor-associated lymphangiogenesis, micro-lymphatic vessel density (MLVD) was determined by quantitative analysis of podoplanin-positive vessels as we described before [12]. Four-micrometer thick tissue sections were collected and incubated with the monoclonal antibodies against podoplanin (rabbit monoclonal, 1:200; Abcam).The lymphatic vessels were counted in three most vascularized fields in non-consecutive sections in a block of tissue and were recorded as total number per unit area. The MLVD count of 4 to 12 vessels composed the low MLVD group according to the statistically greatest inverse proportion to the overall survival in accordance with the mean MLVD (12.59 vessels), whereas the others (≥13 vessels) were subjected to the high MLVD group.

### Statistical analysis

The correlation between MLVD category and the clinicopathologic factors were evaluated using the chi-square test. The cut-off value for the MLVD was set by satisfying both the greatest range-dependent correlation with the overall survival and the mean MLVD value. Survival curves were estimated using the Kaplan–Meier method. For identification of independent prognostic factors, the Cox proportional hazard model was used in the univariate and multivariate analyses. All statistical analyses were performed using the SPSS (version 19.0). OR stands for odds ratio that attempts to quantify the strength of the association between different variables. Statistical significance was defined as *p* <  0.05.

## Results

### Clinicopathological characteristics

Of the 106 patients enrolled in the study, all patients had undergone liver resection and none received preoperative adjuvant chemotherapy or radiation therapy. The clinicopathological characteristics of the cohort are described in Table 1. The cut-off range, > 9 ng/ml (≥9.1 ng/ml), for AFP was set according to the hospital’s serum positivity for the AFP, which was also the indicative value for hepatocellular carcinoma in some hospitals [13]. Serum CA19–9 was also stratified according to the cut-off value of abnormal elevation (≥35 U/ml), which is a frequently used cut-off value for ICC in Chinese centers, such as Sun Yat-sen University Cancer Center [14]. 42 patients (39.6%) were infected with hepatitis B virus (HBV) before the operation through laboratory tests. Alpha-fetoprotein (AFP), one of the specific tumor markers for hepatocellular carcinoma, was below the normal level in most patients with ICC (*n* = 78, 73.6%), while CA19–9 was above the cut-off value in majority of patients (*n* = 73, 68.9%) included in the study. During the operation, number of patients with largest tumor diameter over than 50 mm and multiple tumors nodes was 61 (57.5%) and 17 (16.0%), respectively. Presence of vascular invasion, lymph node metastasis and perineural sheath infiltration, which represented tumor progression, were found in 28, 48, 14 patients, respectively. The median follow-up of total patients was 22.1 months (maximum, 118 months). Tumor recurrence occurred in 72 patients by the end of follow-up.

**Table 1:** Clinicopathological characteristics of 106 patients with ICC enrolled in the study

Clinicopathological Characteristics	Value (%)
Median age (years)	60.0 (range, 35–82 years)
Gender	
Male	62 (58.5%)
Female	44 (41.5%)
HBV infection	
Absent	64 (60.4%)
Present	42 (39.6%)
Preoperative AFP (μg/l)	
< 9	78 (73.6%)
≥ 9	28 (26.4%)
Preoperative CA19–9 (U/ml)	
< 35	33 (31.1%)
≥ 35	73 (68.9%)
Tumor size (mm)	
< 50	45 (42.5%)
≥ 50	61 (57.5%)
Tumor number	
Single	89 (84.0%)
Multiple	17 (16.0%)
Vascular invasion	
Absent	78 (73.6%)
Present	28 (26.4%)
Lymph node metastasis	
Absent	58 (54.7%)
Present	48 (45.3%)
Histologic differentiation	
Well or moderate	52 (49.1%)
Poor	54 (50.9%)
Distribution	
Unilobar	41 (38.7%)
Bilobar	65 (61.3%)
Perineural sheath infiltration	
Absent	92 (86.8%)
Present	14 (13.2%)
TNM stage	
I	33 (31.1%)
II	15 (14.2%)
III	10 (9.4%)
IV	48 (45.3%)
Tumor recurrence	
Absent	34 (32.1%)
Present	72 (67.9%)

### Tumor-associated lymphangiogenesis in ICC

**Fig. 1:**
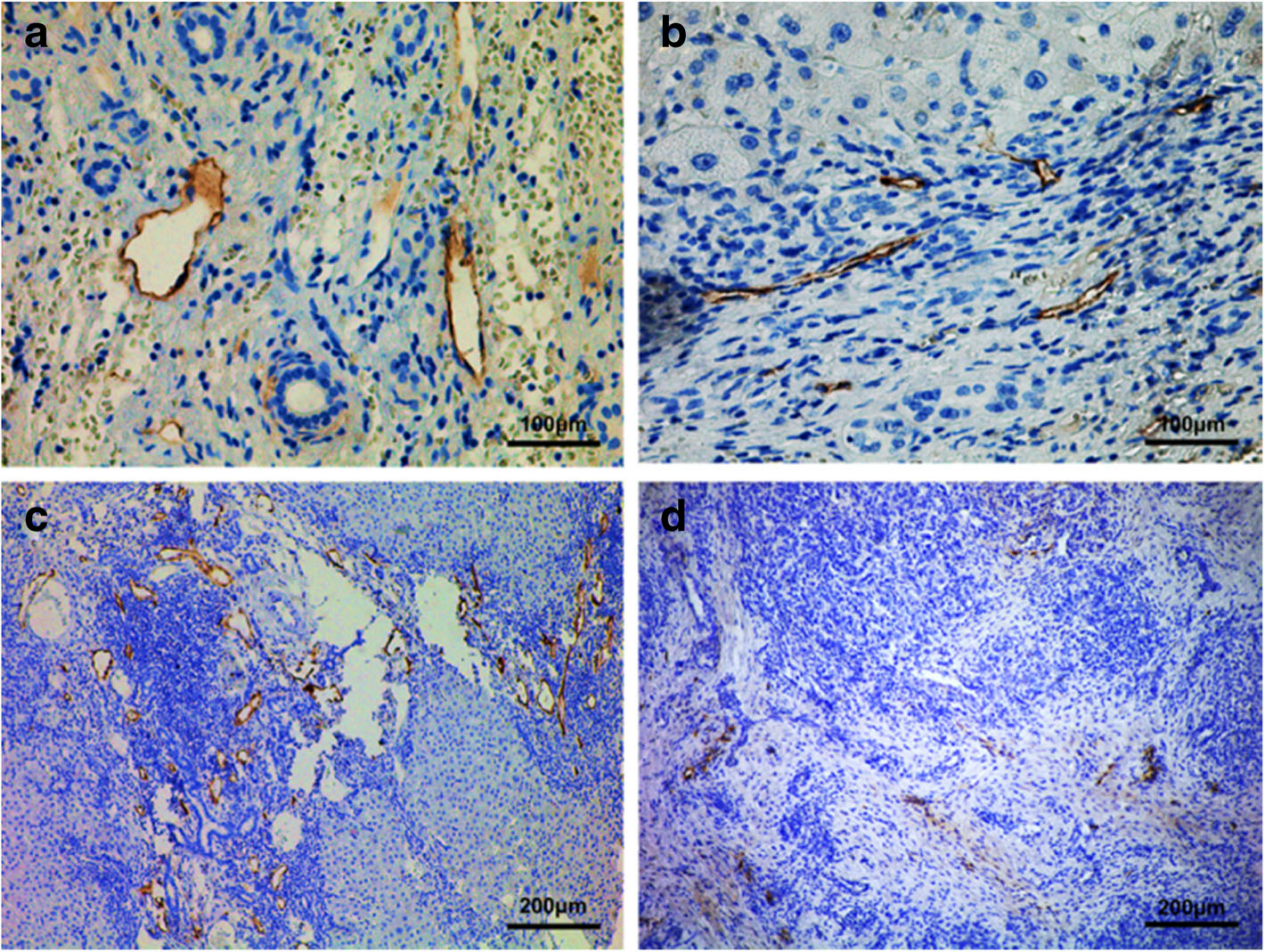
Immunohistochemical staining of podoplanin for evaluation of tumor-associated lymphangiogenesis in ICC. Podoplanin-positive lymphatic vessels exhibited both open and collapsed lumen, respectively (panel **a**, **b**; scale bar = 100 μm). Representative tissue sections for high (**c**) and low (**d**) MLVD (scale bar = 200 μm)

**Fig. 2:**
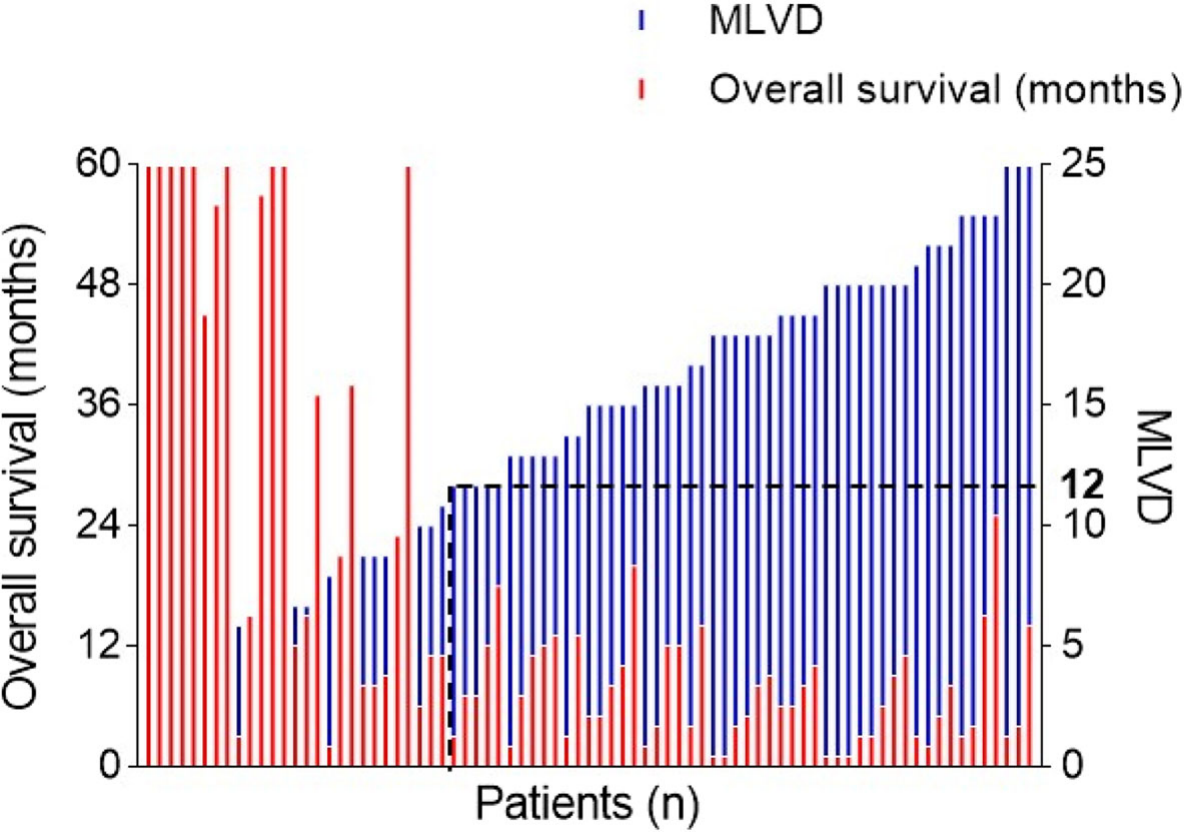
Spearman’s correlation test between MLVD and the overall survival. Spearman’s correlation test indicated that the range 4 to 12 MLVDs is a distinctive range with significant inverse proportion to the overall survival (*P* <  0.0001). However, the range 13 to 25 showed no significant proportion to the overall survival (*P* = 0.4791), indicating that all patients involved in this range obtained unfavorable prognosis regardless increase of the MLVD. Patients censored before 5 years of follow-up investigation were not included

Podoplanin-positive lymphatic vessels were detected within the tumors exhibiting both open and collapsed lumina in 106 ICC samples (Fig. 1a-d). In the Spearman’s correlation test for range-dependent MLVD and survival, the greatest *p* value summary (****; *p* <  0.0001) was initially identified in a range of 4 to 12 (r = − 0.6589; 95% confidence interval [CI], − 0.8229 to − 0.3936), whereas the range 13 to 25 showed no significant correlation (r = − 0.1058; 95% CI, − 0.3814 to 0.1871; *p* = 0.4791; Fig. 2 and Table 2). In addition, the calculated MLVD was 12.59 ± 6.10 lymphatic vessels (range, 4 to 25 vessels). Therefore, the impact of the tumor-associated lymphangiogenesis on outcomes and prognosis, patients were divided into the high (> 12 vessels) and low (≤12 vessels) MLVD groups.

**Table 2:** Spearman’s correlation test for stratification of prognostic subtypes according to the MLVD

MLVD	n (%)	r (95% CI)	P (two-paired)	*P* value summary
4 to 4	1 (1.3)	NA	NA	NA
4 to 5	7 (8.9)	0.2041 (NA)	0.5714	NS
4 to 6	11 (13.9)	−0.4077 (−0.8166 to 0.2736)	0.1013	NS
4 to 7	16 (20.3)	−0.4604 (−0.7847 to 0.0619)	0.0456	*
4 to 8	19 (24.1)	−0.5844 (− 0.8255 to − 0.1631)	0.0086	**
4 to 9	23 (29.1)	−0.6729 (− 0.8531 to − 0.3494)	0.0004	***
4 to 10	26 (32.9)	−0.6035 (− 0.8074 to − 0.2709)	0.0011	**
4 to 11	27 (34.2)	−0.6182 (− 0.8124 to − 0.3005)	0.0006	***
4 to 12	32 (40.5)	−0.6589 (− 0.8229 to − 0.3936)	< 0.0001	****
4 to 13	37 (46.8)	−0.6647 (− 0.8169 to − 0.4260)	< 0.0001	****
4 to 14	39 (49.4)	−0.6619 (− 0.8119 to − 0.4299)	< 0.0001	****
4 to 15	44 (55.7)	− 0.6436 (− 0.7930 to − 0.4212)	< 0.0001	****
4 to 16	48 (60.8)	− 0.6377 (− 0.7838 to − 0.4248)	< 0.0001	****
4 to 17	50 (63.3)	− 0.6220 (− 0.7709 to − 0.4085)	< 0.0001	****
4 to 18	56 (70.9)	− 0.6630 (− 0.7915 to − 0.4784)	< 0.0001	****
4 to 19	60 (75.9)	−0.6456 (− 0.7759 to − 0.4624)	< 0.0001	****
4 to 20	68 (86.1)	−0.6752 (− 0.7897 to − 0.5152)	< 0.0001	****
4 to 21	69 (87.3)	−0.6811 (− 0.7931 to − 0.5247)	< 0.0001	****
4 to 22	72 (91.1)	−0.6789 (− 0.7895 to − 0.5256)	< 0.0001	****
4 to 23	76 (96.2)	−0.6149 (− 0.7411 to − 0.4466)	< 0.0001	****
4 to 24	76 (96.2)	−0.6149 (− 0.7411 to − 0.4466)	< 0.0001	****
4 to 25	79 (100)	−0.5966 (− 0.7256 to − 0.4271)	< 0.0001	****

Note: Patients censored before 5 years of follow-up were excluded. *p<0.05, **p<0.01, ***p<0.001, ****p<0.0001

Abbreviations: *MLVD* microlymphatic vessel density *CI* confidence interval. *NA* not applicable. NS, not significant

### Correlation between MLVD and clinicopathological characteristics in ICC

To determine the correlation between the degree of tumor-associated lymphangiogenesis and clinicopathological characteristics, we performed a further statistical analysis. The results of clinicopathological analysis are described in Table 3. Patients’ gender and preoperative tumor markers of AFP & CA19–9 were not significantly different between patients with “high MLVD” or “low MLVD”. In contrast, patients with “low MLVD” were more likely to be infected with HBV preoperatively (29 out of 56 vs. 13 out of 50; *p* = 0.007). In addition, tumors with “high MLVD” showed more frequently lymph node metastasis (32 out of 50 vs. 16 out of 56; *p* <  0.001) and higher level of TNM stage (40 out of 50 vs. 18 out of 56; *p* <  0.001) compared with “low MLVD” tumors. Moreover, patients with “high MLVD” developed tumor recurrence more frequently than patients with “low MLVD” (49 out of 50 vs. 23 out of 56; *p* <  0.001). Other clinicopathological parameters, including tumor size, distribution, number of tumor nodes, vascular invasion, histological differentiation and perineural sheath infiltration were not significantly different between both groups. Further, the MLVD value between patients with different TNM stages was compared as shown in Table 4. It showed that patients with higher level of TNM stage revealed significantly higher MLVD value compared with lower level of TNM stage (15.19 ± 5.70 vs. 9.46 ± 5.05, *p* <  0.001).

**Table 3:** Association between micro-lymphatic vessel density (MLVD) and clinicopathological characteristics of the patients with ICC

Clinicopathological Characteristics	MLVD Analysis		
	Low MLVD	High MLVD	*p* value
No. of patients	56	50	
Age (years)			
< 60	35 (62.5%)	21 (42.0%)	0.035*
≥ 60	21 (37.5%)	29 (58.0%)	
Gender			
Male	30 (53.6%)	32 (64.0%)	0.277
Female	26 (46.4%)	18 (36.0%)	
HBV infection			
Absent	27 (48.2%)	37 (74.0%)	0.007*
Present	29 (51.8%)	13 (26.0%)	
Preoperative AFP (μg/l)			
< 9	40 (71.4%)	38 (76.0%)	0.594
≥ 9	16 (28.6%)	12 (24.0%)	
Preoperative CA19–9 (U/ml)			
< 35	19 (33.9%)	14 (28.0%)	0.511
≥ 35	37 (66.1%)	36 (72.0%)	
Tumor size (mm)			
< 50	27 (48.2%)	18 (36.0%)	0.204
≥ 50	29 (51.8%)	32 (64.0%)	
Tumor number			
Single	50 (89.3%)	39 (78.0%)	0.114
Multiple	6 (10.7%)	11 (22.0%)	
Vascular invasion			
Absent	41 (73.2%)	37 (74.0%)	0.921
Present	15 (26.8%)	13 (26.0%)	
Lymph node metastasis			
Absent	40 (71.4%)	18 (36.0%)	< 0.001*
Present	16 (28.6%)	32 (64.0%)	
Histologic differentiation			
Well or moderate	29 (51.8%)	23 (46.0%)	0.552
Poor	27 (48.2%)	27 (54.0%)	
Distribution			
Unilobar	24 (42.9%)	17 (34.0%)	0.350
Bilobar	32 (57.1%)	33 (66.0%)	
Perineural sheath infiltration			
Absent	49 (87.5%)	43 (86.0%)	0.820
Present	7 (12.5%)	7 (14.0%)	
Tumor recurrence			
Absent	33 (58.9%)	1 (2.0%)	< 0.001*
Present	23 (41.1%)	49 (98.0%)	
TNM stage			
I/II	38(67.9%)	10(20.0%)	< 0.001*
III/IV	18(32.1%)	40(80.0%)	

*p< 0.05 defined as statistical significance

**Table 4:** Relationship between micro-lymphatic vessel density (MLVD) and TNM stage in patients with ICC

	Cases	MLVD (mean ± SD)	*p* value
TNM stage			
I/II	48	9.46 ± 5.05	*< 0.001*
III/IV	58	15.19 ± 5.70	

### Analysis of prognostic factors for overall and recurrence-free survival in ICC

We further investigated specific clinicopathological variables predicting overall and recurrence-free survival after hepatic resection of ICC. The results of the univariate and multivariate analysis for overall and recurrence-free survival were described in Tables 5, 6, 7 and 8. In the univariate analysis for overall survival, MLVD, lymph node metastasis, and TNM stage were found to be significantly associated with overall survival (*p* <  0.001, *p* = 0.003, *p* <  0.001, and *p* <  0.001, respectively). In the multivariate analysis, MLVD (*p* <  0.001) and tumor size (*p* = 0.021) were determined as independent prognostic factors for overall survival after hepatic resection of ICC. For recurrence-free survival, MLVD was also identified as an independent risk factor through univariate and multivariate analysis (*p* <  0.001 and *p* <  0.001, respectively). In addition, though preoperative HBV infection, tumor size, number of tumor nodes, lymph node metastasis and TNM stage were also associated with recurrence-free survival in the univariate analysis (*p* = 0.009, *p* = 0.004, *p* = 0.049, *p* <  0.001 and *p* <  0.001, respectively), only tumor size (*p* = 0.010) revealed independent prognostic significance for recurrence-free survival.

**Table 5:** Univariate analysis of prognostic factors for overall survival in ICC

Variable	Univariate analysis			
	Category	Median survival	*p* value	Confidence interval
Age	< 60 vs. ≥60	24.1 vs. 19.9	0.394	0.767–1.961
Gender	Male vs. Female	19.3 vs. 26.1	0.074	0.957–2.554
HBV infection	Absent vs. Present	20.2 vs. 25.1	0.118	0.411–1.106
Preoperative AFP (μg/l)	< 9 vs. ≥9	19.5 vs. 29.4	0.466	0.469–1.414
Preoperative CA19–9 (U/ml)	< 35 vs. ≥35	25.1 vs. 20.8	0.075	0.952–2.849
Tumor size (mm)	< 50 vs. ≥50	28.4 vs. 17.5	0.003*	1.312–3.631
Vascular invasion	Absent vs. Present	24.9 vs. 14.4	0.062	0.976–2.695
Lymph node metastasis	Absent vs. Present	30.7 vs. 11.8	< 0.001*	1.916–5.215
Tumor number	Single vs. Multiple	23.6 vs. 14.4	0.201	0.815–2.646
Histologic differentiation	Well/Moderate vs. Poor	23.1 vs. 21.2	0.561	0.719–1.838
Perineural sheath infiltration	Absent vs. Present	22.6 vs. 19.0	0.615	0.608–2.321
Distribution	Unilobar vs.Bilobar	23.2 vs. 21.4	0.127	0.894–2.457
TNM stage	I/II vs. III/IV	34.3 vs. 12.0	< 0.001*	2.189–6.403
MLVD	Low vs. High	34.8 vs. 7.9	< 0.001*	3.963–12.620

*p< 0.05 defined as statistical significance

**Table 6:** Multivariate analysis of prognostic factors for overall survival in ICC

Variable	Multivariate analysis			
	Category	Odds ratio	Confidence interval	*p* value
Tumor size (mm)	< 50 vs. ≥50	1.899	1.121–3.217	0.017*
Lymph node metastasis	Absent vs. Present	1.116	0.493–2.526	0.791
TNM stage	I/II vs. III/IV	1.602	0.618–4.150	0.332
MLVD	Low vs. High	5.005	2.569–9.752	< 0.001*

*p< 0.05 defined as statistical significance

**Table 7:** Univariate analysis of prognostic factors for recurrence-free survival in ICC

Variable	Univariate analysis			
	Category	Median survival	*p* value	Confidence interval
Age	< 60 vs. ≥60	19.6 vs. 16.3	0.254	0.827–2.049
Gender	Male vs. Female	16.7 vs. 20.0	0.608	0.711–1.793
HBV infection	Absent vs. Present	14.8 vs. 23.0	0.009*	0.319–0.847
Preoperative AFP (μg/l)	< 9 vs. ≥9	15.3 vs. 25.9	0.658	0.527–1.499
Preoperative CA19–9 (U/ml)	< 35 vs. ≥35	22.0 vs. 16.3	0.122	0.897–2.504
Tumor size (mm)	< 50 vs. ≥50	25.0 vs. 12.9	0.004*	1.258–3.321
Vascular invasion	Absent vs. Present	20.2 vs. 12.0	0.322	0.782–2.116
Lymph node metastasis	Absent vs. Present	27.4 vs. 6.8	< 0.001*	1.747–4.538
Tumor number	Single vs. Multiple	19.9 vs. 8.3	0.049*	1.002–3.064
Histologic differentiation	Well/Moderate vs. Poor	18.0 vs. 18.1	0.727	0.689–1.706
Perineural sheath infiltration	Absent vs. Present	14.5 vs. 18.6	0.960	0.522–1.980
Distribution	Unilobar vs.Bilobar	20.0 vs. 16.8	0.169	0.867–2.269
TNM stage	I/II vs. III/IV	31.0 vs. 7.3	< 0.001*	2.047–5.665
MLVD	Low vs. High	30.7 vs. 3.9	< 0.001*	3.442–10.079

*p< 0.05 defined as statistical significance

**Table 8:** Multivariate analysis of prognostic factors for recurrence-free survival in ICC

Variable	Multivariate analysis		
	Category	Odds ratio (Confidence interval)	*p* value
HBV infection	Absent vs. Present	0.654 (0.386–1.107)	0.114
Tumor size (mm)	< 50 vs. ≥50	2.006 (1.180–3.410)	0.010*
Tumor number	Single vs. Multiple	0.888 (0.486–1.623)	0.700
Lymph node metastasis	Absent vs. Present	0.982 (0.446–2.160)	0.963
TNM stage	I/II vs. III/IV	1.748 (0.715–4.271)	0.221
MLVD	Low vs. High	3.762 (2.000–7.077)	< 0.001*

*p< 0.05 defined as statistical significance

### Impact of MLVD on overall and recurrence-free survival in ICC

**Fig. 3:**
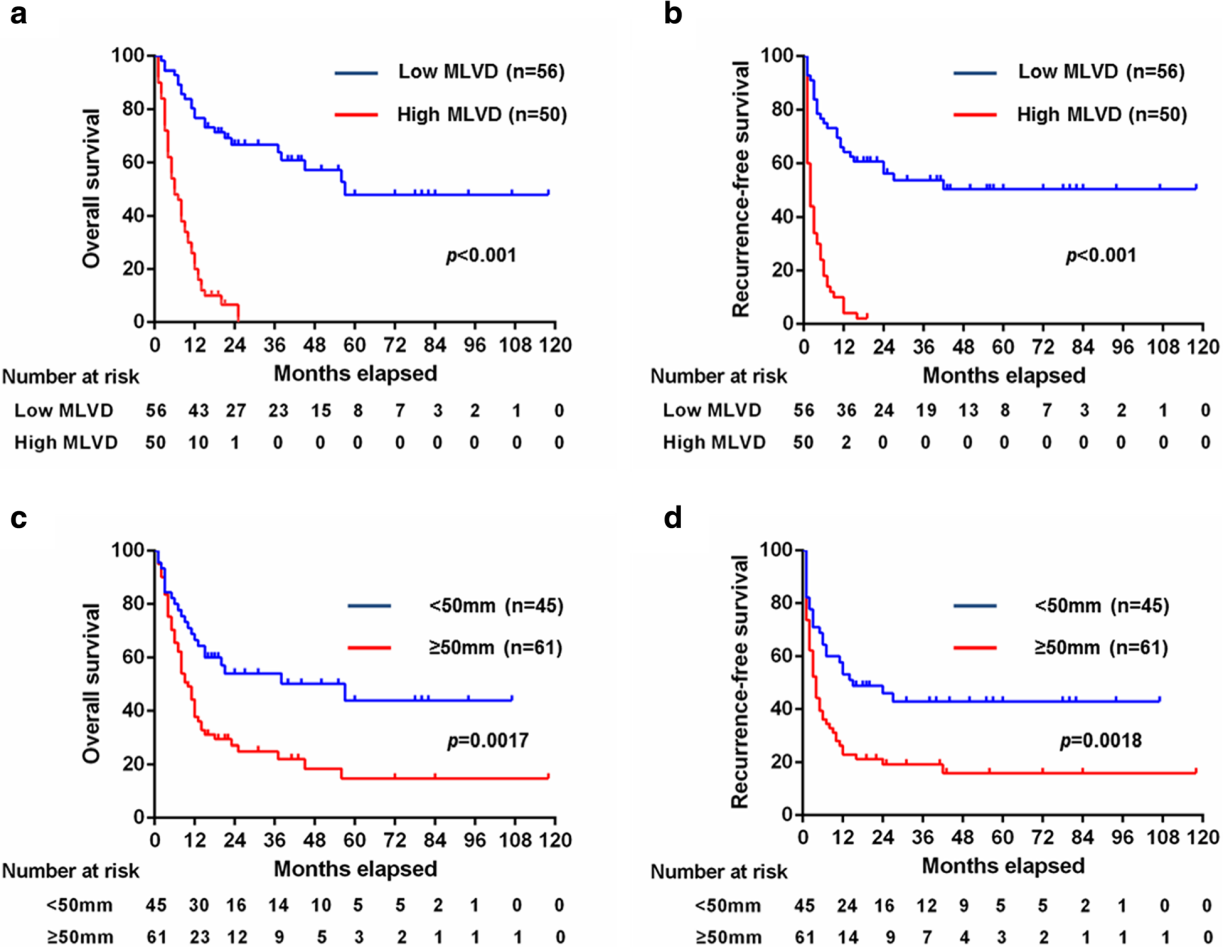
Impact of different level of tumor-associated lymphangiogenesis and tumor size on overall and recurrence-free survival. (a) The overall survival of the high MLVD group (*n* = 50) was significantly worse than the low MLVD group (*n* = 56; *p* < 0.001). (**b**) The recurrence-free survival of the high MLVD (n = 50) was also significantly unfavorable than the low MLVD group (n = 56; *p* < 0.001). (c) Overall survival according to tumor size category revealed that patients with tumors ≥50 mm (*n* = 61) had worse outcomes than those with < 50 mm (*n* = 45; *p* = 0.0017). (d) Patients with tumors ≥50 mm (n = 61) also had much worse recurrence-free survival compared with those with < 50 mm (n = 45; *p* = 0.0018)

Since the abundance of MLVD revealed independent prognostic significance for both overall and recurrence-free survival, we further analyzed the influence of MLVD on survival through Kaplan-Meier method. The 1-, 3-, and 5-year survival of patients with “low MLVD” (*n* = 56) was 76.8, 66.8 and 48%, respectively. In contrast, the “high MLVD” group (*n* = 50) displayed 1-, 3-, and 5-year survival of only 20, 0 and 0%, respectively, (*p* <  0.001), which was much worse than the “low MLVD” group (Fig. 3a). For recurrence-free survival (Fig. 3b), the 1-, 3-, and 5-year survival patients of “low MLVD” was 64.3, 53.8 and 50.4%, respectively, as compared with 4.0, 2.0 and 2.0%, respectively, in patients with “high MLVD” (*p* <  0.001).

In addition to the abundance of MLVD, tumor size is also one of the risk factors for survival of patients with ICC based on the univariate and multivariate analysis. The 1-, 3-, and 5-year survival of patients with tumors< 50 mm (*n* = 45) was 66.7, 54.1 and 44%, respectively, as compared with 37.7, 24.9 and 14.8%, respectively, in patients with tumors≥50 mm (*n* = 61; *p* <  0.01; Fig. 3c). Furthermore, the recurrence-free survival of patients with tumors≥50 mm was also significantly inferior to patients with tumors< 50 mm, with 1-, 3-, and 5-year survival of 23.0, 19.2, and 16.0%, in contrast to 53.3, 42.9 and 42.9%, respectively (*p* <  0.01; Fig. 3d).

## Discussion

ICC is a highly malignant neoplasm with a dismal prognosis due to insufficient evident symptoms at the early stage [15]. Up to date, radical hepatic resection remains the only ideal curative treatment for ICC. However, only a minority of patients with ICC are candidates for radical surgical approaches, with a median survival of 6.3 to 16 months [16]. Lack of appropriate medical approaches for ICC calls for further studies on its clinical and biological characteristics. Frequent lymph node metastasis is one of the major hallmarks of ICC that has been identified as a significant prognostic predictor [17, 18], while tumor-associated lymphangiogenesis in prognosis of ICC remains further confirmation.

In the present study, quantitative analysis of MLVD was performed by counting lymphatic vessels stained with podoplanin. Previously, studies of lymphatic vessels and dissemination have been limited due to lack of specific lymphatic markers. Vascular endothelial growth factor receptor-3 (VEGFR-3) was formerly considered as a marker for lymphangiogenesis. However, VEGFR-3 has also been shown to be expressed in blood vessel endothelium and cancer cells [19, 20]. In recent years, some specific markers, including LYVE-1 and podoplanin, have been identified. However, LYVE-1 has also been found to express on the endothelium of blood vessels [21]. In contrast, podoplanin is expressed specifically in lymphatic endothelium and does not exist in blood vasculature [22]. Therefore, podoplanin was chosen to evaluate tumor-associated lymphangiogenesis in the ICC specimens. Moreover, although not described in the present study, lymphatic vessels in adjacent healthy tissues were also evaluated, which ranged from 0 to 5. These vessels seem to play normal biological roles in the propulsion of the lymph circulation considering its no association with metastasis and prognosis of ICC. In addition, high expression of tumor-associated lymphangiogenesis was found to be closely associated with lymph node metastasis and recurrence, suggesting that tumor cells of ICC are likely to migrate along tumor-associated lymphatic vessels into lymph nodes, thus facilitating recurrence of the tumor that leads to poor prognosis. These findings are in line with several previous studies that reported association between lymphatic vessels and lymph node metastasis in mass-forming tumors [23–25].

In addition, it is noteworthy that patients with low expression of MLVD seemed to be more likely to be infected with HBV preoperatively (*p* = 0.007), which was identified as a prognostic factor for recurrence-free survival (*p* = 0.009). HBV infection has been considered associated with an increasing incidence of ICC in East Asia, where HBV is endemic [26, 27]. However, several studies found that HBV infection may be a favorable factor in patients with ICC. Zhou et al. [28] suggested that the survival of HBV-associated ICC patients was better due to inhibited tumor invasiveness. Wu et al. [29] regarded HBV infection as a favorable factor because of early detection of the unexpected tumor during chronic liver disease follow-up. On the contrary, Ahn et al. [30] reported that 37 HBV-positive and 255 HBV-negative ICC patients showed no significant difference in their outcomes. Since the distribution of HBV infection in ICC was found to vary worldwide, the different results may be attributed to patients enrolled from various regions and different stages of tumor upon diagnosis. Thus, our results require validations in populations from different regions.

Recently, prophylactic lymph node dissection has been considered as a benign factor that might improve the prognosis of the patient with ICC [31]. A study from Japan rendered that the wide dissection, including the regional and para-aortic lymph nodes, along with hemihepatectomy was found to be curative in the first 5-year survivor with periductal-infiltrating ICC [32]. Since tumor-associated lymphangiogenesis is an important independent prognostic factor that deteriorates survival by promoting lymph node metastasis, intraoperative biopsy to evaluate extent of tumor-associated lymphangiogenesis may support decision for performance lymphadenectomy.

Moreover, tumor-associated lymphangiogenesis may serve as one of the novel therapeutic targets of ICC, of which the VEGF-C/VEGF-D-VEGFR3 axis is considered a major driver of tumor-associated lymphangiogenesis [33]. In addition, other growth factors and signaling pathways are also involved in lymphangiogenesis. VEGF-A was found to promote lymphangiogenesis and lymph node metastasis in a mouse fibrosarcoma xenograft model [34]. Platelet-derived growth factor B also induced tumor lymphangiogenesis and promoted lymph node metastasis in mice, but this could not be prevented using a VEGFR3-targeted antibody [35]. While, transforming growth factor-β (TGF-β) has been reported to be a negative regulator of lymphangiogenesis in cancer. Inhibition of endogenous TGF-β signaling in mouse xenograft models of pancreatic adenocarcinoma induced lymphangiogenesis [36]. Based on these findings, various tyrosine kinase inhibitors targeting VEGF, EGRF or FGF receptors such as sorafenib, sunitinib, regorafenib, etc. are under clinical trials for different types of tumor [37–39]. Since clinical trials and reports about antitumor therapy targeting lymphangiogenesis in ICC are still rare, the results of our study may provide a novel view of treatment for ICC.

This study provides a great deal of information about the critical influence of tumor-associated lymphangiogenesis on prognosis of hepatic resected patients with ICC. However, there are a few underlying limitations that remain to be confirmed. Firstly, limited number of patients may cause bias of the results, which could be improved by additional large-scaled studies. Secondly, the involved patients were local malignancies within the liver, patients with advanced and metastatic tumors may result in different outcomes. Lastly, all the involved patients were from Eastern countries, further confirmations by patients from other regions are needed.

## Conclusion

High extent of tumor-associated lymphangiogenesis is significantly associated with lymph node metastasis and recurrence of the tumor in ICC. MLVD, a indicative factor for the extent of tumor-associated lymphangiogenesis, was found to be an independent prognostic factor for overall and recurrence-free survival in patients with ICC after resection. Evaluation of tumor-associated lymphangiogenesis by intraoperative biopsy seems promising to possess a great potential to improve survival outcomes by providing guidance to patient selection who may benefit from lymphadenectomy. We call for future large-scale prospective trials to verify our results for general application in real-world clinical medicine.

## Abbreviations

AFP: Alpha-fetoprotein
CA19–9: Carbohydrate antigen 19–9
HBV: Hepatitis B virus
ICC: Intrahepatic cholangiocarcinoma
MLVD: Micro-lymphatic vessel density
